# Quality Measurement as a Path to High Quality Care: Comment on "Quality and Performance Measurement in Primary Diabetes Care: A Qualitative Study in Urban China"

**DOI:** 10.34172/ijhpm.2023.7884

**Published:** 2023-09-25

**Authors:** John C. Matulis III, Rozalina G. McCoy

**Affiliations:** ^1^Division of Community Internal Medicine, Geriatrics and Palliative Care, Department of Medicine, Mayo Clinic, Rochester, MN, USA; ^2^Division of Endocrinology, Diabetes & Nutrition, Department of Medicine, University of Maryland School of Medicine, Baltimore, MD, USA; ^3^Department of Epidemiology and Public Health, University of Maryland School of Medicine, Baltimore, MD, USA; ^4^University of Maryland Institute for Health Computing, Bethesda, MD, USA

**Keywords:** Quality, Quality Measures, Diabetes, Population Health, Primary Care, Healthcare Delivery

## Abstract

A rigorous evaluation of the implementation of a diabetes quality measure implementation program across community healthcare clinics in Shanghai, China, where both quality measurement and primary care delivery are relatively recent but centrally supported, identified important concerns about the meaningfulness, feasibility, and accuracy of quality measures that are relevant to all quality measurement programs. These include the importance of stakeholder involvement in measure development and implementation, the need to select measures that accurately and reliably reflect care quality, the link between incentives for improved performance and data manipulation, the necessity for scientific credibility and practical feasibility of the measure, and the assurance that measure performance can be impacted by those being evaluated. In addition to elaborating on these aspects of quality measurement, we also discuss the need for quality measures that are balanced across established domains of quality, are not burdensome to participants, and are transparent, parsimonious, nimble, and oriented around continuous evaluation and improvement.

 The increasing incidence and prevalence of diabetes is a threat to advances in life expectancy and quality of life. In 2019, 463 million people were living with diabetes worldwide (9.3% of the world adult population), with the prevalence expected to rise to 700 million people (10.9% of the world adult population) by 2045.^1^ The morbidity, disability, and mortality associated with diabetes stem from its many complications: acute complications of severe hypoglycemia and hyperglycemia (ie, diabetic ketoacidosis, hyperglycemic hyperosmolar state) and chronic complications of cardiovascular disease, kidney disease, neurological complications, amputations, retinopathy/blindness, and among others. The risk of these complications can be mitigated through optimal glycemic control. Accordingly, health systems, professional societies, and regulatory bodies worldwide have focused extensively on systems and processes which support timely and effective control of hyperglycemia on both individual and population levels. Quality measurement is essential and foundational to all such efforts.

 That one might endeavor to measure the care that is provided, as well as the outcomes that flow from that care is intuitive. For over a century, the concept of measuring different aspects of care, sharing those measurements with patients, and allowing patients to select “high quality” physicians, has been considered a fundamental component of building a better healthcare system. Pioneers such as Ernest Codman began measuring healthcare outcomes in the 1910s,^2^ while the concept of assessing and measuring the discrete structures, processes, and outcomes of healthcare through the lens of the Donabedian model was described in the 1960s.^3,4^ In today’s healthcare environment, quality measurement is pervasive, both in the United States and around the world.

 Despite the widespread acceptance of quality measurement, important questions remain on what actually comprises high quality care, including how individual and societal preferences may influence the perception of quality, and how structural and social determinants of health, among many other factors, need to be considered when measuring “high quality care.”^4^ As such, contemporary quality measurement can miss what is important to many patients, clinicians, and healthcare systems, and fail to deliver actionable information to stakeholders. Even when clarity can be achieved in developing measures that reflect shared societal values, measurement can be disproportionately weighted towards measures of clinical effectiveness and neglect other important dimensions of quality including timeliness, efficiency, patient centeredness, and equity of the care provided.^5^ In an attempt to remedy these shortcomings, measure developers have created more measures, more complex measures, measures which may not be grounded in strong evidence, and measures that may not actually be under the purview of the healthcare system or the physician to improve.^6^

 The largest number of people with diabetes — nearly a quarter of the world population with diabetes — live in China, though just under half are aware that they have this condition.^1^ Cognizant of the need for quality measurement to improve diabetes management and health outcomes, as well as its potential downsides, Rasooly and colleagues conducted a large scale evaluation of the implementation of diabetes quality measurement across community healthcare centers delivering primary care in Shanghai, China.^7^ Specifically, they conducted in-depth interviews with endocrinologists, primary care physicians, community healthcare center managers, patients, and policy makers across two tertiary hospitals (which deliver specialty care and where patients historically received the majority of their diabetes care) and four community healthcare centers. They focused specifically on a core set of three diabetes quality measures set at the national level and used by the Shanghai Health Commission since 2009: (1) the health management rate, reflecting the percent of residents in the community healthcare center’s catchment area with diabetes who are treated by the community healthcare center; (2) the standardized management rate, representing the proportion of patients with diabetes treated by the community healthcare center who are seen there at least quarterly; and (3) the glycemic control rate, reflecting the percent of patients with diabetes treated by the community healthcare center who have fasting blood glucose <7 mmol/L. These measures are reported to the municipal health commission and used for large (potentially exceeding 50% of their total compensation) performance-based bonuses to physicians, nurses, and public health practitioners and promotion opportunities for community healthcare center and district leaders.

 Rasooly and colleagues share important learnings from their efforts to understand the front-line team members’ experience with quality measurement, which can be informative to stakeholders across different settings, populations, and regulatory environments. First, because these measures were developed and implemented without front-line clinician input — as most quality measures are — there were concerns about their appropriateness, feasibility, and usefulness. Some measure developers seek out stakeholder feedback and engagement, for example incorporating technical expert panels and having a public comment period, but even then, more transparency is needed to showcase the specific impact stakeholders had on the final quality metric. Pre-implementation field testing of quality measures, with publicly available evaluation results, can help ensure feasibility and utility. Second, while quality measures informed and prioritized practice initiatives to improve performance, these efforts seemed more focused on improving numeric *measurement of quality*, rather than the actual *quality of care* and downstream health outcomes as perceived by patients, physicians, and other stakeholders. Stakeholder engagement not only during the measure development process but throughout its life course can help ensure that the measure is meeting its intended objectives.

 Third, to sufficiently incentivize behavioral change, quality measures were designed to have a substantial impact on both physician reimbursement and community healthcare center managers’ career advancement. These incentives — while effective at motivating practice priorities and initiatives around these measures — also raised concerns about coerciveness, data manipulation, and “gaming” the system. Indeed, it was this concern for data manipulation vis-à-vis inclusion of healthy individuals as having diabetes that led to the decision to discontinue the health management rate measure. This is to be commended, as quality measures are rarely examined for unintended consequences or undesired implementation and are even more rarely de-implemented for these reasons. Additionally, quality control mechanisms with verification of reported data for the remaining measures were introduced, with punitive consequences for false reporting. While participants in the study felt that this did not entirely eliminate false reporting, it did make it more difficult, though also introduced additional administrative burden to measurement and reporting. Again, seeking stakeholder feedback after measure implementation proved invaluable.

 Fourth, the quality measure must be aligned with contemporary clinical care processes and scientific evidence. For example, while quality measurement called for attaining fasting blood glucose <7 mmol/L, the preferred means of monitoring glycemic control in patients with existing diabetes is hemoglobin A_1c_ (HbA_1c_) and not fasting blood glucose.^8^ Yet, HbA_1c_ testing was neither covered by insurance nor included in the measure, hindering implementation of the measure. For many people living with diabetes – particularly those treated with intensive insulin therapy – alternative markers of glycemic control such as time in range may be even more appropriate. Allowing flexibility to be responsive to contemporary scientific evidence and best practices would make quality measures to be evidence-based and patient-centered but may not be feasible for standardized measures used for public reporting and/or reimbursement (as opposed to internal quality improvement). Conversely, by making glucose-lowering medications available for longer periods and at lower cost at community healthcare centers than hospitals, measure attainment was made more feasible. Fifth, attaining the quality measure must be within the control of the individuals and entities held accountable for them. For example, while primary care physicians and community healthcare centers were accountable for diabetes quality measure attainment, many patients continued to bypass them in favor of hospital-based specialists. Broadening the eligible pool of responsible entities to include all clinicians who assume primary responsibility for the patient’s care — irrespective of their affiliation — would help support diabetes management on population scale. Sixth, the quality measures were viewed as disconnected from how patients experience their own health and what matters to them; engaging patients and their care partners as stakeholders in the quality measure development process can improve patient engagement with the healthcare system and their self-management. Finally, there were concerns about a rigid or militarized organizational culture that precluded innovation and optimization of care delivery at all levels, which may not be readily modifiable but underscores the importance of local culture and customs on health and healthcare delivery.

 In their study, Rasooly and colleagues also shared several broadly generalizable findings useful to policy makers and measurement developers across different settings. First and foremost, they found bi-directional communication between measure developers and program participants to be critically important, so that challenges to the validity and implementation of the measure can be iteratively identified, communicated, and addressed. Withdrawal of a measure, as in the case of the health management rate, speaks to the open dialogue necessary for success between measure developers and those ultimately accountable to those measures. They also highlight the value of parsimony in measurement development, demonstrating that selecting a small number of meaningful measures that can be iteratively improved allows for greater clarity and engagement with program participants. The authors also identified that both patient and physician experiences are important to attaining durable stakeholder engagement. Finally, insights into the impact of public reporting of rankings on performance and motivation were shared.

 There are important limitations to this work. Many patients in Shanghai continued to receive diabetes care through hospital systems instead of community health centers and these hospital-based practices may have had a more limited opportunity to introduce innovative solutions to improve performance. The challenge of understanding the locus of control for measures parallels the challenges faced in other settings where primary care practices are liable for quality of care that may be delivered by others. While some new policies and interventions for improving performance of these measures were referenced, little detail was provided on what was done, why, and how successful these interventions were. There are also significant concerns about the generalizability of these findings to other settings. Most of the clinics appeared to be in urban settings, and we do not know how these findings may translate to rural settings or to clinics with fewer available resources. Social determinants of health, which have a profound effect on diabetes management,^9^ were largely absent from this analysis. While the authors did carefully consider the patient’s experience of care within the community healthcare clinic setting, the gap between what patients and physicians consider high quality care and the patient facing discussion on how measurement systems could be further improved were not explored. Aligning measures between patient and physician perceptions of high quality care is a significant challenge, and an area where further research is needed. Strategies which have been implemented include integrating patient perspectives in the measurement development process,^10^ balancing clinically oriented measures with patient reported outcomes.^11^

 These findings should prompt measurement developers and stakeholders to reimagine the development and implementation of quality measurement to ensure that both the measures and the measurement process are responsive to the needs of all stakeholders in the diabetes care journey (Table).

**Table:** Common Concerns and Mitigation Strategies in Quality Measurement

**Quality Measure Development Question**	**Concern**	**Mitigation Strategy**	**Example**	**Shanghai Health Commission Program: Description and Validity**
What are we measuring?	Is the evidence linking the quality measure to the desired outcome robust? Does measuring the proposed indicator allow for detection of clinically meaningful changes in health outcomes or other aspects of care that are meaningful to stakeholders?	GRADE^12^ or GRADE-like recommendations are transparently applied to measure selection process.	There is a robust evidence base in support of lowering HbA_1c_ levels to reduce risks of chronic diabetes complications, though the exact threshold of HbA_1c _should be informed by the patient’s clinical complexity and a countermeasure for hypoglycemia should be considered.	Composite measure of healthcare utilization and glycemic control. Utilization measures (appt attendance) have not been linked to improved outcomes. Fasting glucose measurement is inferior to HbA_1c_ in assessing diabetic control.
Why is this specific indicator being measured?	Is the quality measure intended to support internal quality improvement efforts, being used to empower patients in selecting their healthcare provider and/or organization, or to encourage healthcare organizations to focus on this indicator?	Involvement of Patient-Family Advisory councils and multi-group stakeholder consortiums can collectively assert the primary rationale for initiating the measurement program. This rationale should guide all subsequent modifications to the program.	A multi-stakeholder consortium, consisting of payors, physicians, patients, and community members, uses a consensus building process to agree on a prioritized set of diabetes measures that are realistic to improve, understandable to patients and other stakeholders, and represent a community health priority.	The purpose of the program is to encourage physicians to focus on this indicator. As done in this work, input from a consortium of diverse stakeholders can improve the relevance and value of the measurement program.
What are the requirements for participating in the measurement program?	Does collecting and reporting the quality measure require investment apart from routine practice? What is the burden on patients, physicians, and healthcare systems?	Resources for collecting, storing, and reporting data should be made available to all participants if significant additional investment is required to report that measure.	Accepted measurements should be feasible for health systems to collect, analyze, and report without requiring substantial investment in electronic or personnel resources.	Practices would need to register, enter, and update the records of patients participating in this program. The burden and feasibility of data collection was not directly assessed.
Who should be responsible for improving performance on the measure?	Is the individual provider responsible for improving this measure or should this measure be the responsibility of the organization, the community, or the health plan?	Measures should be assigned to entities with operational control over their attainment. There should be clarity and transparency about who is charged with improving performance on that measure.	Primary care physicians should not be the sole responsible entity for quality measure attainment if they are not solely responsible for delivering diabetes care and attaining measure outcomes.	Primary Care Physicians and community-based clinics. Specialist and other care were not included, and often, patients attributed to a physician were not receiving care through them.
What are the financial consequences of meeting or not meeting the metric?	A single metric may have disproportionate impact on the physician’s or practice’s revenue and even on the financial viability of that practice, resulting in undue pressure to meet the measure at all costs. Conversely, a measure can have limited impact on income or operations of the practice, limiting its impact on performance.	Financial incentives need to be large enough to be meaningful, yet not so large that poor performance could impact viability.	A health system participating in the measurement program can select available measures with different levels of risk and reward. Risk and reward trade-offs need to be meaningful but not coercive.	Substantial financial incentives and penalties for the practice and the individual physician were tied to this measure. Based on reports of false reporting and “gaming the system,” the degree of financial incentive through this program needs to be re-considered.
What are the burdens and opportunity costs of the measurement, tracking, and reporting of the quality measure?	Is there a measurement and reporting burden to the provider and system? Is there a substantial opportunity cost with prioritizing this measure over other measures?	The number of quality measures must be limited to allow for a seamless/automated measurement system; the measures must reflect the priorities of the selected stakeholders.	Health systems, in collaboration with relevant stakeholders, can select from a narrow number of vetted measures which they find feasible, meaningful, and impactful to their communities.	Quality measurement programs have been reported as parsimonious, and this is the main measure primary care physicians are responsible for. The specific degree of burden or opportunity cost is not directly reported in this work.
Could this measure worsen health disparities?	Prioritizing certain measures could result in penalization of practices caring for disadvantaged populations and promote selective inclusion and exclusion of patients.	Stratified reporting of the measurement based on social determinants of health and health disparities should be considered.	Diabetes outcomes are stratified by socioeconomic status, access to transportation, availability of social services and other social determinants of health. Efforts to address social determinants of health and improvements in measure attainment within subgroups of populations are also considered.	Yes, as diabetes control and healthcare utilization are strongly predicated on Social Determinant of health. This was not directly reported in this work.
What are the unintended or undesired consequences of pursuing the attainment of the quality measure?	Focus on a particular quality measure could result in overtreatment, non-individualized treatment goals and targets, purposeful selection of patients, manipulation of documentation and/or data, and detrimental impact on patient trust and the clinician-patient relationship.	Balancing measures should be implemented across different domains; counterbalance measures are needed.	HbA_1c_ target measures are coupled with hypoglycemia reporting. Measures are individualized based on patient’s health status and life expectancy. Measures of efficiency, timeliness of care, patient experience and safety are incorporated within the measurement program.	Unknown. Considerations of measures of patient cost, rates of hypoglycemia, and healthcare access could be considered in future analyses.

Abbreviation: HbA_1c_, hemoglobin A_1c_.

 In addition, all relevant stakeholders (payors, patients, clinicians, other healthcare team members, health system leaders, and quality experts) need to be identified and their input on an optimal measurement approach needs to be carefully considered. This level of engagement and contribution must go beyond the Technical Expert Panels and public comment periods used by the US Centers for Medicare and Medicaid Services for quality measure development, and should seek to understand the healthcare needs, practice priorities, and implementation challenges faced by people responsible for and affected by measure attainment. Second, to ensure that quality measures truly measure quality, they must capture domains of quality beyond effectiveness and include measurements of the efficiency, safety, timeliness, patient-centeredness, and equity of the care provided.^13^ The value compass,^14^ summarized in Figure, is a tool that quality measure program developers can use to help guide their balance of measures, and to assure one domain isn’t under or over-represented. The domains, including the clinical effectiveness of the care provided (in Rasooly et al captured via glycemic control measure discussed above), the experience of care (captured, but not quantified), patient functional outcomes (not captured) and the costs and utilization of care (incompletely captured via utilization measures). Measure developers and evaluators can use the value compass in considering both quality measurement program design and evaluation, carefully considering the implications of measures related to cultural expectations around the experience of care, and addressing factors relevant to costs of care in healthcare systems outside of the United States where the value compass was developed. Third, while we are calling for a set of balanced measures, the number of measures must be limited to a narrow set of actionable measures with a strong evidence base for measuring and supporting quality.^13^ Indeed, a robust and balanced measure set can be derived from as few as four measures.^14^ Fourth, quality measures should be periodically reexamined to ensure that they are still meeting their objectives of improving quality without unintended consequences or manipulation. While countermeasures and data integrity initiatives can reduce the risks of undesired consequences, introduction of such efforts need to be weighed against the concerns for measurement burden. The discontinuation of the Shanghai Health Commission’s health management rate measure is a good example of responsive measure redesign, while policing efforts to prevent data manipulation and fraud can easily outweigh their benefits.

**Figure:**
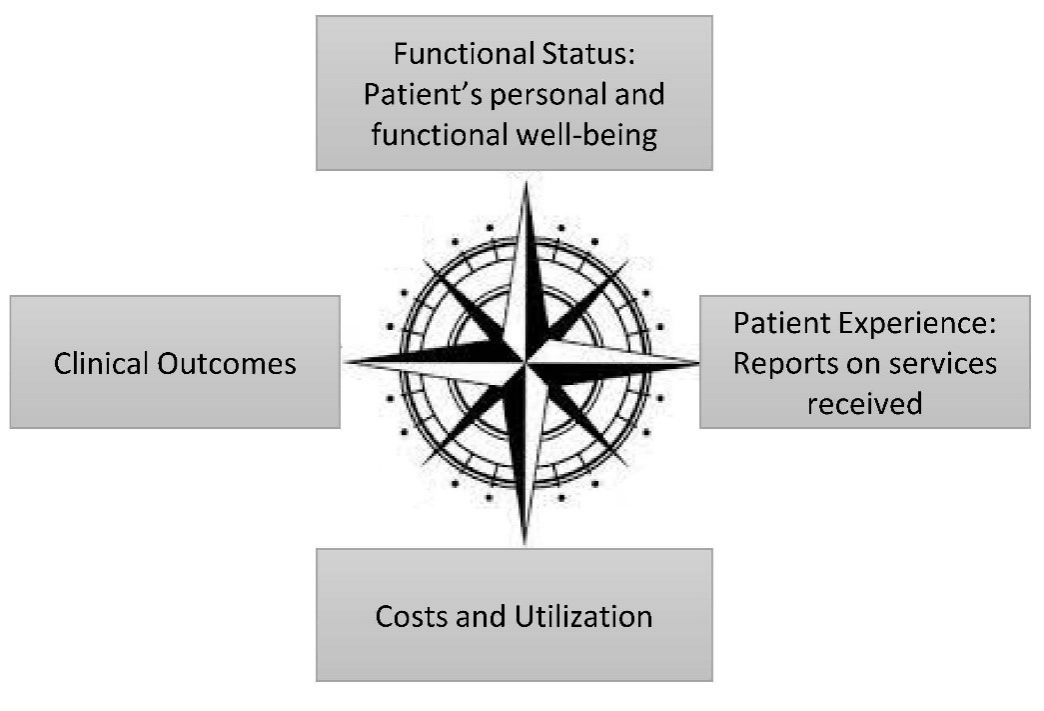
The Clinical Value Compass.

 We have previously examined the themes describing what healthcare stakeholders perceive to be an ideal quality measure.^15^ Engaging a multi-disciplinary panel of clinicians, pharmacists, nurses, researchers, and representatives of both public and private health plans, we identified three themes and core value systems that underpin an ideal quality measure of diabetes management: promoting individualized, evidence-based and equitable care; balancing autonomy and prescriptiveness in clinical decision-making; and ensuring an accurate, reliable and practical quality measurement. These themes resonate with the findings of Rasooly and colleagues.

 Quality measurement, tracking, and reporting has been fundamental to nearly all efforts to improve diabetes care quality. Despite a proliferation of quality measures, evidence that measurement programs truly improve the quality of diabetes care and, more importantly, patient health, quality of life, and costs of care is scarce. The work of understanding, engaging with, and responding to the needs of different stakeholders in evaluating the quality of care is perhaps the most important challenge of 21st century healthcare delivery. It is therefore important to critically evaluate quality measure development, implement, and maintenance — as Rasooly and colleagues have done — to ensure that measures truly improve care delivery and health outcomes.

## Ethical issues

 Not applicable.

## Competing interests

 In the last 36 months, Rozalina G. McCoy has received support from the National Institute of Diabetes and Digestive and Kidney Diseases (NIDDK), National Institute on Aging (NIA) of the NIH, the Patient Centered Outcomes Research Institute (PCORI), and the National Center for Advancing Translational Sciences (NCATS). She also serves as a consultant to Emmi® (Wolters Kluwer) on developing patient education materials related to diabetes.

## Funding

 This effort was funded by the NIDDK of the National Institute of Health (NIH) grant number K23DK114497 (McCoy).

## Disclaimer

 Study contents are the sole responsibility of the authors and do not necessarily represent the official views of the NIH.
